# *“It’s Just Addictive People That Make Addictive Videos”*: Children’s Understanding of and Attitudes towards Influencer Marketing of Food and Beverages by YouTube Video Bloggers

**DOI:** 10.3390/ijerph17020449

**Published:** 2020-01-09

**Authors:** Anna Elizabeth Coates, Charlotte Alice Hardman, Jason Christian Grovenor Halford, Paul Christiansen, Emma Jane Boyland

**Affiliations:** Department of Psychological Sciences, University of Liverpool, Liverpool L69 7ZA, UK; cah@liverpool.ac.uk (C.A.H.); j.c.g.halford@liverpool.ac.uk (J.C.G.H.); prc@liverpool.ac.uk (P.C.); eboyland@liverpool.ac.uk (E.J.B.)

**Keywords:** food, beverage, HFSS, influencer marketing, YouTube, children, understanding, attitudes, qualitative, focus group

## Abstract

Exposure to influencer marketing of foods and beverages high in fat, sugar, and/or salt (HFSS) increases children’s immediate intake. This study qualitatively explored children’s understanding of, and attitudes towards, this marketing, to elucidate potential mechanisms through which exposure affects behavior. In six focus groups (*n* = 4) children (10–11 years) were shown a YouTube video featuring influencer marketing of an HFSS product. Inductive thematic analysis identified six themes from children’s discussions of this marketing: (1) YouTubers fill a gap in children’s lives, (2) the accessibility of YouTubers increases children’s understanding of their actions, (3) influencer marketing impacts all—the influencer, the brand, and the viewer, (4) attitudes towards influencer marketing are most affected by a YouTuber’s familiarity, (5) YouTuber influencer marketing is effective because they are not ‘strangers’, (6) children feel able to resist influencer marketing of HFSS products. Children had an understanding of the persuasive intent of this marketing, and although most were sceptical, familiar YouTubers elicited particularly sympathetic attitudes. Children felt affected by influencer marketing of HFSS products, but believed they were able to resist it. Beyond theoretical insight, this study adds to the growing body of evidence to suggest children’s exposure to HFSS influencer marketing should be reduced.

## 1. Introduction

Exposure to food and beverages high in fat, salt and sugar (HFSS) is widely acknowledged as a risk factor for the development of obesity and other non-communicable diseases in children [1,2,3]. Systematic reviews highlight the detrimental effects of HFSS product marketing on young people (0–18 years), including increased preferences for, and consumption of, these items [4,5]. They also highlight significant gaps in research to date, notably a lack of qualitative studies exploring how newer forms of digital marketing (e.g., influencer marketing) are received by children [6]. Exploration of children’s understanding of, and attitudes towards, HFSS product marketing in digital media, which are hugely popular with young people [7], could provide insight into how exposure affects behavior.

YouTube is one of the most popular social media platforms with children, despite users being required to be a minimum age of 13 years in order to create an account [7]. Notably, an account is not required for users to view videos on YouTube. In addition, children use parents’ accounts, and fake date of births to create their own accounts, to access account-holder privileges [8]. Approximately 72% of 10–12-year-olds in Australia [9], 85% of 13–17-year-olds in the US [10], and 80% of 5–15-year-olds in the UK [7] use YouTube. Children report valuing the platform for its choice and frequent renewal of video content [7]. Children are frequent viewers of YouTube video bloggers (YouTubers), individuals who create and share videos on YouTube [11], and view this content as a source of entertainment [7,12]. YouTubers can organically feature food and beverage products in their videos [13,14] but often also receive payments or gifts from brands for doing so [15,16,17]. Collaborations between brands and YouTubers (also referred to as ‘influencers’) is a fast-growing marketing technique referred to as ‘influencer marketing’ [17]. Industry spend on this form of marketing is predicted to increase from $500 million per year in 2018 to $5–$10 billion per year in 2023 [18].

Children are frequently exposed to influencer marketing of HFSS food and beverage products and brands on social media [14,19,20,21]. This is a concern given that evidence suggests that exposure, via Instagram [22] and YouTube [23], increases children’s (9–11 years) immediate consumption of HFSS products, compared with exposure to equivalent marketing of non-food products. However, little is known about the theoretical or psychological underpinnings of these effects. Social cognitive theory [24] would assert that these findings are likely due to children viewing influencers as role models to learn appropriate behaviors. Consistent with this theory, research shows that children prefer celebrity-endorsed HFSS products and brands [25,26,27,28]. Alternatively, source credibility theory asserts that if an endorser is perceived to be a credible source of information then consumers will likely develop a positive attitude towards the promoted product [29,30,31]. Source credibility is determined by several factors, including the perceived fit between the marketed product and the endorser, the likeability of the endorser, and the level of risk associated with adopting the endorser’s behavior [32,33]. Studies have yet to fully understand the extent to which children view influencers as role models or as credible sources of information.

Although children’s perception of influencer marketing of HFSS products specifically has not been explored, qualitative research has focused on their understanding of influencer marketing of non-food products [34,35,36]. Findings from these studies demonstrate that compared with other forms of digital marketing (e.g., YouTube pop-up advertisements), children (9 and 12 years) find influencer marketing via YouTubers to be less irritating because it does not interrupt media content, and provides useful product information (e.g., price) [36]. Children also feel that YouTubers promote products that are more relevant [34,35], likely because influencer marketing is embedded in content that children have actively selected to watch [7,12]. In their videos, YouTubers regularly disclose personal details [37] and speak to the viewer directly [38]. Viewers can then communicate with them via YouTube’s ‘comment’ and ‘like’ functions and as a result may feel a sense of familiarity with YouTubers [39]. The literature shows that product recommendations from familiar influencers are trusted more than traditional celebrities [31,40,41,42] or influencers who are less familiar [11,17,43,44]. However, research has not qualitatively explored whether children’s familiarity with YouTubers affects how they receive influencer marketing of HFSS products. Research is needed to explore these views in more detail and to understand whether this may influence children’s understanding of, and attitudes towards, influencer marketing of HFSS foods and beverages.

Persuasion knowledge is the ability to recognise and evaluate the persuasive attempt of advertising [45,46]. Early theories of advertising, such as the persuasion knowledge model, assert that young children (12 years and under), compared with adults, are less able to activate persuasion knowledge and resist the effects of advertising because they have limited cognitive abilities [47,48,49]. However, in relation to digital marketing specifically, qualitative research demonstrates that children (9–11 years) *do* have the ability to activate persuasion knowledge [50]. Nevertheless, persuasion knowledge alone may not be sufficient to protect children against the effects [51,52]. Compared with adults, children have greater reward sensitivity and impulsivity [53], and lack executive control [54]. In addition, children nearing adolescence are also more susceptible than younger children to social appeals and branding [53,55]. Therefore, even with knowledge of persuasive intent, children’s appetitive response can be influenced by HFSS product marketing [22,56,57,58,59]. In order for children to defend against the effects of food and beverage advertising, the food marketing defence model [52] asserts that four conditions must be satisfied: awareness of advertising, understanding of its persuasive intent, and the ability and the motivation to resist. Children’s satisfaction of these conditions has not been qualitatively explored in response to exposure to influencer marketing of HFSS products and may provide insight into how and why children are affected.

Digital marketing techniques, such as influencer marketing, often take the form of non-advertising content and can be difficult for even adults to recognise as advertising [15,60,61,62]. To try to mitigate against deception, regulations in some countries, including the self-regulatory codes in the UK, require that influencers use an advertising disclosure to highlight the commercial nature of this content. It is suggested that products featured in receipt of brand payment should be disclosed with ‘#ad’ [63] and those in receipt of brand gifting with ‘#gifted’ [64]. However, research shows that children (9–11-years) exposed to influencer marketing of a HFSS product, even with the presence of an advertising disclosure, increase immediate intake compared with those in a control group [22]. Qualitative research with children (9 and 12 years) shows that subscribers of a YouTuber viewed their marketing of a beauty product as an indication of competence and popularity, whereas non-subscribers remarked on their incompetence and were critical of the commercialisation of YouTubers in general [36]. Similar qualitative research with adolescents [65] and adults supports these results [31,66]. The scepticism-identification model of advertising [67] asserts that the findings of these studies are likely due to an advertising disclosure triggering two opposing effects on viewers; scepticism about the competency of the source, and identification with the source. A viewer’s scepticism is low and identification high when a viewer has a pre-existing knowledge of the consumer (e.g., when an influencer is followed on social media), however, a viewer’s scepticism is high and identification low when the source is an unfamiliar consumer (e.g., when an influencer is not followed on social media). Research has not yet explored children’s attitudes towards advertising disclosures used in influencer marketing and whether attitudes remain for familiar and less familiar influencers.

Based on the identified gaps in knowledge, the following research questions were formulated for this study: (1) What are children’s perceptions of YouTubers? (2) What is children’s understanding of, and attitudes towards, the techniques used in influencer marketing of HFSS products? (3) What is children’s understanding of, and attitudes towards, the behavioural effects of this marketing? Given that these research questions were focused on children’s subjective viewpoints and experiences, qualitative methods were deemed to be the most appropriate way to capture this information.

## 2. Materials and Methods

### 2.1. Sampling

The study was approved by the University of Liverpool Institute of Psychology, Health and Society Research Ethics Committee (reference: 3995), and data were collected in May 2019. A convenience sample of children (*n* = 24, 12 female) were recruited from schools in Liverpool. Parents were informed of the study by school distribution of study information sheets and completed opt-in consent forms if they were happy for their child to take part. The number of parents who did not provide consent was not recorded due to researcher time constraints. Children were read a child-friendly information sheet and, if willing, gave their assent. All children assented, there were no dropouts, and no repeat focus groups.

The study adopted a qualitative inductive approach to data collection. Six focus groups were conducted with children aged 10–11 years. At this age, children are highly active on YouTube, despite platform age restrictions being set at 13 years [7]. Focus groups are an established method for conducting research with school-age children and provide rich, in-depth data [68,69]. They were chosen over individual interviews because they enable children to be stimulated by the perspectives of others, and to feel less inhibited than they may do in a one-to-one setting [70]. Focus group size (n = 4) and duration (45–55 min) were based on recommendations for conducting qualitative research with children [69,71] and similar previous research [36,65,72].

### 2.2. Materials

#### 2.2.1. YouTube Video Featuring Influencer Marketing

Children were shown a YouTube video that featured influencer marketing of an HFSS product to inform focus group discussions. The video, ‘Nutella Breakfast Party’ (https://www.youtube.com/watch?v=ZyIHfeguqlE) was uploaded to YouTube in 2018 and featured a paid promotion for the HFSS product Nutella chocolate spread (Ferrero S.p.A, Alba, Italy). The video was obtained using the download software KeepVid (https://keepvid.com) and edited using VideoPad video editor (http://www.nchsoftware.com/videopad/index.html). Editing ensured that children were only exposed to the marketing segment (6 min) of the original video (21.11 minutes), meaning they would only share their opinions on the commercial content, and they would be less likely to become bored [69,71]. The original video was created by male YouTuber Alfie Deyes (‘PointlessBlog’) but also frequently featured his girlfriend Zoe Sugg (‘Zoella’), also a YouTuber. At the time of writing, both YouTubers were in their late twenties, were considered by the authors to be a healthy weight and had previously promoted HFSS products on YouTube. The male YouTuber had a subscriber count of approximately 11 million across his three YouTube channels, and the female YouTuber a total of 16 million subscribers across her two YouTube channels. The YouTubers’ viewer demographics are not publicly available but they are known to be popular with children (5–15 years) in the UK [73] and are consistent with those used in previous experimental studies investigating the extent, nature and impact of influencer food marketing [14,22,23]. Notably, the influencer marketing campaign shown was featured in content likely to be viewed by children, and not in content specifically targeted at them (e.g., YouTube Kids app), where self-regulation should prohibit exposure [74]. Both YouTubers primarily upload videos about their day-to-day lives as well as specific interests such as beauty and online gaming. The content of the YouTube video used in this study was considered to be appropriate for children and to appeal to both female and male viewers alike.

The self-filmed video begins with the YouTuber talking to the viewer about how he and his family will be celebrating ‘World Nutella Day’. An advertising disclosure (#ad) is displayed in the bottom right-hand corner of the screen for a duration of 10 s while the YouTuber explains how Nutella approached him ‘to work on a video together’. A competition for viewers to win three large jars of Nutella is then introduced, which viewers can enter by Tweeting or tagging the YouTuber on Instagram with the hashtag ‘#WorldNutellaDay’. The entire video takes place in the YouTuber’s home and features himself, his YouTuber girlfriend and four family members. It includes preparation and consumption of foods made with Nutella (e.g., blueberry and Nutella muffins), with Nutella visible or spoken about throughout. During the video the YouTuber shows the viewers a gift that he received from Nutella (a large padded seat which resembled a jar of Nutella), and he and his girlfriend and family are filmed taking photographs of the foods so that they can be shared on social media. At the end of the video viewer engagement is encouraged by the YouTuber asking his viewers to share recipe ideas, images and videos of how they celebrate World Nutella Day via the hashtag ‘#WorldNutellaDay’, and by tagging the YouTuber on Instagram and Twitter. Viewers are also asked to ‘like’ the video and ‘subscribe’ to the YouTubers’ channel.

#### 2.2.2. Photographs of Influencer Marketing Techniques

Six photographic stills (see Supplementary Materials S1) were created from the YouTube video using the VideoPad video editor. Five captured an influencer marketing technique (e.g., the use of hashtags to increase viewer engagement) and one captured the on-screen advertising disclosure #ad. Each photograph was presented individually during focus groups to probe children’s understanding and attitudes.

#### 2.2.3. Interview Guide

A semi-structured interview guide (see Supplementary Materials—Interview guide) led focus group discussions and was developed by the project team. The semi-structured format is recommended for use with children and allows for deviation and flexibility while ensuring consistency in topics across groups [75]. The guide included non-leading, open-ended questions allowing data collection on children’s perceptions of YouTubers, influencer marketing of an HFSS product, and effects on behaviour. Children were encouraged to comment on each other’s responses [76] to enable conversations about influencer marketing that researcher probing alone may not have achieved.

### 2.3. Procedure

Children were selected for focus groups in friendship pairs (two boys, two girls) by the class teacher. Focus groups were conducted by the lead researcher (A.C.), a female PhD student trained in qualitative research and experienced in conducting research with young children. The study location was in a small room close to the children’s classroom within their school. Children were seated around a table with a laptop computer and no other persons were present. Children were informed that their participation was voluntary and that with their permission the discussion would be audio recorded but that names would be removed when reporting the results. Children were told that the aims of the study were to gather their opinions on YouTubers, and YouTubers who advertise food and drinks. To minimise response bias, they were informed that the study was not a test and there were no right or wrong answers.

Prior to study commencement, no relationship had been established with children and so at the start of each focus group the researcher introduced herself. A series of questions on social media use were administered to build a rapport and to check whether each child was familiar with watching YouTubers, and the general concept of influencer marketing. The interviewer used child-friendly probing techniques (e.g., prompts that used simple wording) and took a neutral position on all topics discussed. Children were then instructed that they would watch a video by the YouTuber Alfie Deyes which featured his YouTuber girlfriend, Zoe Sugg. Most children had heard of the two YouTubers and in general expressed liking their content. No child commented on their dislike for either YouTuber or disclosed that they regularly watched the videos of either one. For those few children who were less familiar with the YouTubers, the interviewer described them as being ‘popular YouTubers’. Children were also informed that this video featured influencer marketing for Nutella chocolate spread. Most children liked Nutella; however, three children disliked the taste or had allergies. Children were then shown the YouTube video and were asked not to talk during viewing as a discussion would take place afterwards. The semi-structured interview guide and photographic stills were used to lead focus group discussions. The interviewer allowed children to speak freely about the influencer marketing campaign, but also about previous encounters with this type of marketing. Field notes were made by the interviewer to inform follow-up questions in future groups. At the end of the focus group, children were asked if there were any further comments they wished to make, debriefed and thanked for their time.

### 2.4. Analysis

Focus groups were audio recorded and transcribed verbatim by the same researcher who conducted the focus groups, A.C. After transcription, each recording was listened to again so that any errors could be corrected. Transcripts were not returned to participants for comment or correction. Transcripts were anonymised and imported into NVivo 12 qualitative data management software (NVivo, Version 12). Inductive thematic analysis was conducted by A.C., allowing codes to be generated from the data itself, rather than being theory driven [77]. Thematic analysis was well-suited to these data because it acknowledges that individuals create meaning from their own experiences (i.e., individual experiences of influencer marketing) as well as the broader social context (i.e., social and environmental determinants of how influencer marketing is interpreted). Transcripts were repeatedly read for familiarisation. Words and sentences were firstly open-coded according to their unit of meaning, resulting in a long list of codes. Data were only coded if they were relevant to the three main research questions (1) children’s perceptions of YouTubers (2) children’s understanding of, and attitudes towards, the techniques used in influencer marketing of HFSS products and (3) children’s understanding of and attitude towards the behavioural effects of this marketing. Notably, although coding was guided by these topics it was imperative that children’s perspectives were dominant during the coding process. Data that were superfluous to the research questions were not coded. Similarities and differences between codes were then identified and were clustered into major categories and subcategories. Comparisons and contradictions were made within and between these categories, allowing for construction of themes, guided by the three main research questions. Participants were not asked to provide feedback on the findings. Feedback on the initial codebook and themes was provided by E.B., and the codebook is available upon request.

## 3. Results and Discussion

Focus group transcripts were analysed to gain a deeper understanding of (1) children’s perceptions of YouTubers (2) children’s understanding of, and attitudes towards, the techniques used in influencer marketing of HFSS products, and (3) children’s understanding of, and attitudes towards, the behavioural effects of this marketing. A total of six themes were developed: (1) YouTubers fill a gap in children’s lives, (2) the accessibility of YouTubers increases children’s understanding of their actions, (3) Influencer marketing impacts all-the influencer, the brand, and the viewer, (4) attitudes towards influencer marketing are most affected by a YouTuber’s familiarity, (5) YouTuber influencer marketing is effective because they are not ‘strangers’, and (6) children feel able to resist influencer marketing of HFSS products.

### 3.1. RQ1: Children’s Perceptions of YouTubers

#### 3.1.1. Theme 1: YouTubers Fill a Gap in Children’s Lives

Despite being younger than YouTube’s minimum age requirement for having an account (13 years), all children had watched videos by YouTubers, and the majority were subscribed to a YouTuber’s channel. YouTubers were valued for their provision of entertainment, information, acceptance and experience, across a diverse range of content. Children’s viewpoints on each of these provisions are now discussed in more detail.

Many children perceived watching YouTubers’ videos as a source of entertainment or as a go-to activity to fill in time—M (girl, aged 11): “If you are bored then you just watch it and it makes you feel better”. For these children, watching YouTubers provided them with positive reward and prevented boredom. In contrast, a small minority of children reported perceiving it as a “waste of time” and found it difficult to comprehend why others would be entertained by watching strangers talk about themselves. These children often expressed a preference for other types of video (e.g., ‘satisfying videos’), likely because this content was considered to better fulfil their needs [78].

YouTubers were also considered as providers of information, F (boy, aged 11): “these YouTubers that play the games, I’m going to get one of those games soon, so I’m trying to see the play through, see how it works.” In this quote, the child refers to using the YouTuber’s content to improve their online gaming skills. However, there was a consensus that there was a YouTuber to support all interests. Their videos were also valued for giving “honest” and “proper” product reviews, and were often consulted to inform future purchase decisions, such as which video game to buy next.

Some children watched YouTubers who had a similar character, lifestyle or interests to themselves—P (boy, aged 11):

“He [YouTuber] is someone I look up to. Most people think negatively about what I think about some stuff, but he doesn’t. I like dinosaurs, he likes dinosaurs. Lots of people think it’s weird that I like dinosaurs. He’s very positive about it and he plays games with them in. I look up to him.”

In the above quote, the child identifies with the YouTuber’s interest in dinosaurs and harnesses it as validation of their own interest. Having the same beliefs and interests as others is important for social acceptance [72] thus, when a child does not receive acceptance from those around them in real life (e.g., family or peers), YouTubers can fulfil this role.

Children also sought out and watched videos from YouTubers whose lives and personalities were different to their own. Many described doing so to gain insight into others’ experiences—L (boy, aged 10): “I’m not remotely the same as them. It’s just interesting to see what they do, and then I go out and compare what I do.” It was frequently claimed that YouTubers get to do ‘cool’ activities, one child, J (boy, aged 10), recalling a YouTuber who: “jumped off the Eiffel tower onto a trampoline.” Parental control and a lack of independent spending power were often blamed by children as reasons for them not being able to partake in such activities themselves, rather they seemed to seek to experience them vicariously through YouTubers.

The purpose that YouTubers served for children was seemingly dependent on the needs of each child, and in turn these needs informed children’s choice of which YouTubers to watch. Children may form strong attachments with YouTubers because they feel reliant on their content to fulfil their needs. Indeed, some likened their dependence on YouTuber’s content as an addiction—K (girl, aged 10): “it’s just addictive people that make addictive videos”, although adults too have described it in this way [31]. The gap that YouTubers fill, which appears to differ for each child, likely plays a role in how marketing by these characters is understood. This concept is further explored in Theme 3.

#### 3.1.2. Theme 2: The Accessibility of YouTubers Increases Children’s Understanding of Their Actions

Overall, YouTubers were viewed to be less famous than traditional celebrities (i.e., movie stars), predominantly because their fame is specific to YouTube, as opposed to across various media. As a result, it was understood that YouTubers live their lives like “normal people”. For example, one child, H (boy, aged 10), stated that movie stars: “might go out in a blacked-out car so that people can’t take pictures of them […] whereas YouTubers just go”. This perception is consistent with previous research [38,79] and is likely why so many children identified with these characters (Theme 1).

Whereas traditional celebrities were understood to have management teams that communicate with fans, YouTubers were assumed to personally respond to their viewers, and may explain why previous qualitative research finds children (9 and 12 years) perceive YouTubers to be accessible [36]. However, like adults [39,80], most children in the current study had only ever experienced one-way interactions with YouTubers—H (boy, aged 10):

“I actually had a clip on my Twitch [live streaming platform], but I didn’t know how to clip it. I was asking them [YouTuber] how, and they didn’t respond. It was so sad.”

Despite a lack of return communication, children may still perceive a level of intimacy with a YouTuber, by being active in their communication with these characters [39]. This was evident in the following quote, as this child had never received a response from their favourite YouTuber, yet they still considered them a friend—L (boy, aged 10): “it’s like sending a text to your best friend”. Adults, too, have described the relationship in this way [37,39]. Although some children reasoned that a physical connection is required for a friendship, they often described their favourite YouTubers as being people they look up to—K (girl, aged 10): “I just really love them”, and whose content they watch regularly (Theme 1).

Familiarity between children and YouTubers appeared to influence judgement of their actions. For example, children’s favourite YouTubers were often understood to be driven by a passion for making videos, whereas less familiar YouTubers were deemed to be more driven by financial incentive, earning money from merchandise, advertising revenue, their own brands, and sponsorship deals—S (girl, aged 11):

“I like Sophie Darcy. She’s a contortionist and her videos are really funny. They’re original […] I think to be a good YouTuber it’s not doing it for the fame and money, it’s doing it because you actually like doing it, and you like filming. She just has that.”

In this excerpt, as well as many others, the actions of the child’s favourite YouTuber are regarded more favourably. Thus, familiarity with a YouTuber may also affect how children perceive the decision to market products in their content, which is discussed in Theme 4.

### 3.2. RQ2: Children’s Understanding Of, and Attitudes towards, the Techniques Used in Influencer Marketing of HFSS Products

#### 3.2.1. Theme 3: Influencer Marketing Impacts all—The Influencer, the Brand and the Viewer

When probing children’s understanding of the influencer marketing techniques used to promote Nutella, opinions were divided on who they best served: the influencer, the brand or the viewer. The perceived benefits for each are detailed below:

##### Influencer Marketing Impacts the Brand

The purpose of influencer marketing was understood by most children as a means to increase awareness and purchasing of Nutella—L (boy, aged 10):

“They (Nutella) paid him to spread the word out. They used a really popular YouTuber. He can spread the word fast […] to his 10 million [subscribers], they might spread it, and then it might go all over the world. Like viral. That gets Nutella loads of money.”

In the above quote the child displays a clear awareness of the sizeable impact that just one YouTuber can have for a brand. The impact is understood to be achieved by the YouTuber increasing viewers’ exposure to the brand, which can be further increased by subscribers sharing this marketing with others (i.e., peer marketing). In the campaign, the YouTuber requests that his viewers share images and recipe ideas via the hashtag #WorldNutellaDay. Although some children felt the YouTuber had a genuine interest in his viewers, others felt their interest served the brand:

Interviewer: “Alfie asks the viewers to share recipes using hashtag World Nutella Day, why?”

R (boy, aged 11): “Probably to get more recipes for Nutella.”

L (girl aged 10): “Or to see what you (the viewer) can do with Nutella, the creations you can make.”

T (boy, aged 11): “I don’t think he would try the recipes.”

R (boy, aged 11): “He’d be like ‘Oh, I like it’ (wink wink).”

Some of those who were sceptical of the YouTuber’s interest believed that YouTubers act like market researchers. They do so by gathering viewers’ ideas, which “might be better than the ones they (the brand/influencer) had”, so that the brand can create better future campaigns. Indeed, advertisements that are targeted, such as those tailored to viewers’ preferences, are found to be particularly impactful [81,82]. In addition, many recognised that viewers would need to purchase Nutella in order to share these images and recipe ideas and so sales of Nutella would increase. When exploring children’s understanding of the competition to win a jar of Nutella, a few believed that it plants the idea of obtaining Nutella, so that when unsuccessful, a viewer would purchase a jar instead. This is perhaps especially likely given that Nutella is inexpensive, as is common for prizes on social media HFSS brand pages [83].

##### Influencer Marketing Impacts the Influencer

Some children felt that YouTubers who collaborate with popular brands like Nutella benefit from this partnership, just as celebrities do from brand endorsements [84]. YouTube videos which feature influencer-brand collaborations were understood to attract subscribers of the brand ‘Nutella fans’ as well as subscribers of the YouTuber, and so would likely gain more views. Additionally, some children were astute in their observation that YouTubers will more likely promote sweet foods than other food types because they are more desired. As a result, these videos will receive better viewer engagement, earning the YouTuber increased income from advertising revenue and potential future collaborations. One child even joked about the idea of a YouTuber promoting a less desirable food type, such as meat—T (boy, aged 11): “If they were promoting meat, for example, people wouldn’t watch it, because it’s just meat.”

##### Influencer Marketing Impacts the Viewer

Although some children believed there were no benefits for viewers exposed to influencer marketing: “It doesn’t work well for us,” consistent with previous research which finds that viewers dislike the commercialisation of YouTube [13,37], many saw some benefits. For example, it was preferred to other forms of digital marketing (e.g., YouTube pop-up advertisements) because it was perceived as a child’s choice to watch this content, as opposed to being forced to watch marketing that interrupts media content—S (girl, aged 11):

“With this type (influencer marketing) you are pressing on it because you want to watch it whereas with pop-up ones, you might not want to watch it.”

Although influencer marketing does in a sense interrupt media content, as a YouTuber’s regular non-commercial content is replaced with commercial content, children did not consider this an interruption. This may be because these children claimed to watch and identify with YouTubers who have similar interests to themselves (Theme 1) and so they would likely only promote products deemed relevant to their interests. Previous research finds that children have a more positive attitude towards advertisements for relevant products compared to non-relevant products [34,35,36,72]. Children also described watching YouTubers’ regular content to gain information and experience (Theme 1) and influencer marketing may serve the same purpose. For example, some children described feeling inspired to recreate the YouTuber’s Nutella recipe ideas shared in the video. Adolescents also value the information provided in influencer marketing [65]. In addition, competitions and discount codes provided during influencer marketing of HFSS products were viewed to particularly benefit children because they enable access to products which are usually restricted due to parental control or a lack of financial means: “If your mum said no, and you have no pocket money, you could have a chance.” Thus, irrespective of whether a YouTuber’s content features marketing or not, the role YouTubers serve may fundamentally be the same.

#### 3.2.2. Theme 4: Attitudes towards Influencer Marketing Are Most Affected by a YouTuber’s Familiarity

Persuasion knowledge of influencer marketing was often expressed as a lack of trust in the YouTuber’s opinion of the promoted product—H (boy, aged 11): “It’s saying that he’s [the YouTuber’s] not doing it because he wants to do it, but he’s doing it because he’s paid to.” Some even assumed that the entire video was scripted and that the YouTuber had no creative input. Children disliked the positive bias that was displayed towards Nutella-H (boy, aged 11): “They (YouTuber) made it look like it was amazing, like it was the best thing they’ve ever had. Like everyone likes it, no one hates it.” Many had the attitude that had the YouTuber not been paid to promote Nutella, he may have been more honest about the possible negative effects of consuming the product too often—J (girl, aged 11): ”They [YouTuber] might warn you to not constantly eat Nutella because if you become addicted to it, you might develop an allergy.” Clues from the YouTuber’s video, such as the YouTuber’s healthy weight status, were often used to support children’s scepticism that they truly like and consume the product regularly.

The YouTuber’s presentation of Nutella, and foods in advertising in general, were judged to be not representative of real life:

H (boy, aged 11): “They [YouTuber] make it nice for the video. When you’re at home you don’t care. You just spread [Nutella] and [gestures big bite].”

J (girl, aged 11): “Like with McDonalds. Their burger ads [adverts] are like, ‘Look at this amazing burger’.”

N (boy, aged 11): “[…] in the adverts you’ll see a perfect burger; you get it from the store, and it looks like a hippopotamus just sat on it [laughs].”

In the excerpt above, there is a clear consensus that marketed foods are made to look more attractive than in reality. One child even displayed an acute understanding that the YouTuber likely consumes Nutella with fruits to make the product appear more appetising and healthier (a technique frequently used in HFSS product marketing [85,86])—J (girl, aged 11): “[…] all the bright colors with the fruit, it’s healthy, but it’s probably not that healthy because of all the Nutella.”

The YouTuber’s celebrations of World Nutella Day were also considered to be an inaccurate reflection of how people would celebrate in real life, one child believing their family might do so by making “a Nutella sandwich”. The foods children described were often fewer and more basic in comparison with the quantity of “fancy” foods shown in the YouTuber’s celebrations. Children recognised that it would take time, effort and money to celebrate as the YouTuber did, which most felt was not attainable to them due to restrictions in their lives (e.g., parental control, etc.). Children reasoned that an accurate representation of how people consume Nutella in real life was likely not shown “because it’s boring” and so would be less entertaining to watch. Although children were sceptical, many also enjoyed watching the YouTuber create and consume foods they were less able to.

Scepticism towards influencer marketing was less prominent when children were asked to consider how they would feel if their favourite YouTuber promoted Nutella:

L (girl, aged 11): “The girl I watch always gives her honest opinion. If she doesn’t like it, she’ll say so.”

T (girl, aged 11): “Yeah, that’s like the people that I watch. They might try something, and they actually give their proper opinion. But not all people do that.”

Children were often more favourable in attitude towards familiar YouTubers. These findings are consistent with previous qualitative research with children (9 and 12 years) [36] and are supportive of the scepticism-identification model of advertising [67]. This model asserts that disclosure of advertising has two opposing effects on viewers; scepticism about the competency of the source, and identification with the source.

Regulations in the UK require that influencers use an advertising disclosure to highlight products featured in receipt of brand payment ‘#ad’ [63] or brand gifting ‘#gifted’ [64]. Consistent with the opinions of children and adolescents in previous qualitative research [50,65], many children accepted that products are endorsed for financial gain—K (girl, aged 10): “they [YouTuber] are trying to make money, make a living, and enjoy their life.” In fact, the presence of an advertising disclosure was argued by some to be of little significance as exposure to a food product or brand is still achieved—L (girl, aged 10): “even if it wasn’t an advert, you are still posting it online. You’re still advertising.” YouTubers were said to have a similar attitude, one child recalling how one joked that a branded beverage featured in their content was not featured due to financial gain—H (girl, aged 11): “She [YouTuber] got a Coca Cola and said, ‘hashtag not sponsored’. It just made me laugh.” Such declarations can be confusing [87]. Relatedly, children were confused by YouTubers who essentially do the opposite and make content look like influencer marketing, through the use of hashtags and brand names, in order to attract future brand deals. Thus, it is not surprising that children misinterpret influencer marketing in YouTube videos [83].

These findings have interesting implications for the regulation of advertising disclosures. Not only do many influencers fail to comply with the rules [20,88,89], but many children in the current study did not consider advertising disclosures to have any real significance in determining their attitude towards the promoted product. This is likely why research shows advertising disclosures have no protective impact on children’s (9–11-years) immediate intake when featured during influencer marketing of an HFSS product [22]. As suggested in Theme 2, children were less sceptical of marketing by YouTubers whom they feel they know well. This is a concern because children’s greatest exposure to marketing (including for HFSS products) will inevitably be from their favourite YouTubers, courtesy of their more frequent viewing of this content.

### 3.3. RQ3: Children’s Understanding Of, and Attitudes towards, the Behavioural Effects of This Marketing

#### 3.3.1. Theme 5: YouTuber Influencer Marketing Is Effective Because They Are Not Strangers

Influencer marketing of HFSS products was considered to be particularly effective because YouTubers are familiar, whereas in other forms of marketing (e.g., television advertisements) endorsement is often provided by strangers—K (girl, aged 10): “I think YouTubers are a bit better because you might know the person on YouTube, with adverts you don’t know who’s doing it.” These findings are consistent with source credibility theory, which asserts that if a source is deemed to be credible, then viewers will develop a positive attitude towards the product [29,30,31]. On the other hand, some children believed that well-known endorsers were not important for this marketing to be effective, and simply recommending a food to a person would likely convince them to try it. M (boy, aged 10): “If I told someone there was a new food called Nutella, they might try it. Even if I told a random stranger, they would try it.” The attitude shared in this quote supports the work of researchers who claim that electronic word of mouth (eWOM) is an influential marketing technique [90,91,92,93]. Not only is influencer marketing akin to an eWOM recommendation, given that children are trusting of YouTubers who are familiar (Theme 4), but it also promotes genuine eWOM recommendations to be shared by encouraging viewers to share this marketing (Theme 3).

Some children thought that influencer marketing is likely effective because viewers will wish to imitate the YouTuber’s behavior—M (boy, aged 11): “If they [viewer] know a famous person likes it […] they want to be like him, so they’ll think ‘Oh, I’ll get some Nutella’.” This understanding supports the claims of social cognitive theory [24] and research that shows that children wish to copy celebrity lifestyles [94,95]. Advertising that conveys popularity and status from consuming food brands may help viewers to establish their own identities [53], just as watching YouTubers with similar interests and beliefs can (Theme 1).

It was noted that a drawback of influencer marketing is that an influencer may be disliked, and so their endorsement of an HFSS product may actually negatively affect the brand—H (boy, aged 11): “They [viewer] might hate Alfie Deyes [YouTuber], so they might think, ‘He’s advertising it. I, from now on, hate Nutella.” Although this is a valid point, supported by a meta-analysis of the effectiveness of celebrity endorsement [42], it is unlikely that children watch YouTubers that they do not like. Unlike celebrity endorsement campaigns on television, which are viewed by many different types of people, influencer marketing is more likely only viewed by those that admire or who have an interest in the influencer.

Children’s beliefs about the likely effects of influencer marketing are consistent with advertisers’ increased spend in this area [17,18] and as result of this fast-growing industry children are frequently exposed to influencer marketing of HFSS food and beverage products [14,19,20,21].

#### 3.3.2. Theme 6: Children Feel Able to Resist Influencer Marketing of HFSS Products

Many children displayed a reasonably comprehensive understanding of the persuasive intent of influencer marketing (Theme 3), consistent with previous qualitative research which explored children’s (9 and 12 years) perception of influencer marketing [36]. This may indicate that earlier theories of advertising, such as the persuasion knowledge model [47] which asserts that young children (12 years and under) are less able than adults to activate persuasion knowledge and so resist the effects, are perhaps outdated. However empirical research shows that advertising awareness alone has no protective effect on children’s appetitive response to digital marketing of HFSS foods [56,57,58,59] or more specifically influencer marketing of these foods [22,23]. This is likely because children under the age of 12 are unlikely to apply advertising knowledge while being exposed to an advertisement, unless they are overtly made aware of the persuasive intent [51]. Consistent with these findings, many children in the current study believed they were affected by exposure to the influencer marketing campaign for Nutella, and commonly referenced experiencing a physiological reaction—H (boy, aged 11): “My mouth’s watering, I’m so hungry.”

The food marketing defence model claims that in order for children to resist the effects of food marketing, four conditions must be satisfied: awareness of advertising, understanding of its persuasive intent, and the ability and the motivation to resist [52]. Some children believed that they had the ability to resist the YouTuber’s promotion of Nutella and that exposure would affect others but not themselves—S (boy, aged 11): “I couldn’t really care less to be honest. It’s only Nutella. Some people will be like ‘Oh my God, my favourite YouTuber said this, so I need to do it’.” Others, however, felt that strategies were required to deal with this marketing. For example, one child spoke of a coping strategy developed specifically to deal with the effect of watching YouTube videos that feature HFSS foods—K (girl, aged 10):

“If I watch a food video, the trick is to get an apple, or some sort of food with you, and eat the apple really slowly, so you don’t run out in the middle of the video, then go ‘Oh, I want that food [advertised]’. You just eat that food, and then you don’t want that food [advertised].”

In the excerpt, the child displays a conscious *motivation* to resist the effect of hunger, cued from seeing foods marketed online. However, her *ability* to resist the effect is questionable. Although she opts to consume a healthy food and considers this to be a strength of her strategy, exposure to the food cue still encourages consumption of calories. Notably, this was only one child reporting such a coping strategy. Food decisions made by children are asserted to be usually less motivated by health than adults [96], which is likely why the majority of children reported no such strategy. Another coping strategy reported to help children resist the effect of influencer marketing of HFSS products was to unsubscribe to YouTubers’ channels, and so avoid future exposure. However, given the connection that most children feel towards these individuals, and their enjoyment of watching this content, this seems unlikely to be widely adopted.

## 4. General Discussion

The current study found that YouTubers seem to be an invaluable tool for children in terms of their provision of entertainment, information, social acceptance and experience. These provisions were valued irrespective of whether YouTubers’ content explicitly features marketing or not. Most children were attuned to the persuasive intent of techniques used in influencer marketing, and although most had sceptical attitudes towards this content, many were more forgiving and trusting of their favourite YouTubers. In fact, favourite YouTubers were accepted for endorsing products for financial gain, rendering advertising disclosures largely insignificant. Most children felt that HFSS products marketed in this way would likely be effective but felt that they were able to resist

Despite suggestions from previous research that children will talk less in a group setting due to peer pressure [69], children in the current study appeared relaxed and were comfortable challenging each other’s viewpoints of influencer marketing. However, the study did have some limitations. Firstly, to avoid children becoming bored, children were only shown the marketing content from the original video. This focused attention on marketing is not how this content is viewed in real life, where it is typically embedded in noncommercial content [97]. Relatedly, it is possible that demand characteristics (e.g., attention drawn to advertising through interviewer questioning) may have impacted children’s responses, leading them to be more focused on the persuasive intent of the content rather than their enjoyment of content itself. Therefore, the findings from this study are in regard to children’s overt attitudes and projections of their vulnerability to marketing. Indeed, previous research shows that children under 12 are unlikely to spontaneously apply this knowledge when confronted with engaging and entertaining marketing [51]. Secondly, not all of the children liked Nutella and so these children may have underestimated their understanding of the marketing impact. Thirdly, children’s previous liking of the YouTuber was not measured, and as discovered in the analysis, likely affected responses [98]. However, children were encouraged to discuss previous experiences of food marketing by their favourite YouTubers to mitigate this issue.

The aim of this qualitative research was to provide novel insight into children’s attitudes and understanding of influencer marketing of HFSS products through social media. In order to draw generalisable conclusions, future research should adopt quantitative research methods to empirically assess whether children’s attitudes towards and understanding of this marketing impacts eating-related behaviors. Future qualitative research could explore how children perceive influencer marketing of healthier foods, for which there is a lack of evidence of impact [23]. Research could also explore how food and beverage cues which are not explicitly declared as advertising are received by children, which is how most food cues are presented in YouTuber videos [14].

## 5. Conclusions

Advertising budgets of global HFSS food and beverage brands enable them to be creative in how they market their products, which when consumed in excess are detrimental to health. For example, in the UK in 2017 over £300 million was spent on advertising HFSS products, compared to £16 million on advertising fruit and vegetables [99]. This study indicates that YouTubers are valued highly by children predominantly because they are viewed to fulfil their needs. The actions of children’s favourite YouTubers, including their decision to promote products in their content, are looked upon more favourably than those who are less familiar. This is a concern given that children’s greatest exposure to marketing (including for HFSS products) will inevitably be via these individuals due to their more frequent viewing of this content. Media literacy programmes (e.g., teaching children about the persuasive intent of advertising) are likely not necessary, as children had a reasonable understanding of the purpose of influencer marketing. Nevertheless, children valued the information shared and were entertained by influencer marketing, particularly if the YouTuber was valued for such provisions in their regular content. Such positive attitudes towards marketing are in stark contrast to children’s attitudes towards other marketing techniques, which are generally experienced as intrusive and disliked. This study offers practical insight from those who are most likely impacted by HFSS product marketing, children, and has important implications for the ongoing societal and political debate about children and advertising. Although children felt able to resist HFSS products marketed in this way, evidence would suggest otherwise [22,23]. The current regulation does not appear adequate in protecting children against exposure to this marketing [100] and there is an obligation for governments under the human rights law to do so [101,102]. Social media platforms should also ensure that accurate user demographic data (e.g., data that reflects the actual age of a user) is collected. A combined effort between policymakers, social media platforms, brands, advertising agencies and influencers is required.

## Supplementary Materials

The following are available online at https://www.mdpi.com/1660-4601/17/2/449/s1, Figure S1: Photographic stills of influencer marketing techniques featured in the YouTuber’s video, Interview guide.
