# Supplementary material for: “It’s Just Addictive People That Make Addictive Videos”: Children’s Understanding of and Attitudes towards Influencer Marketing of Food and Beverages by YouTube Video Bloggers
Source: Int J Environ Res Public Health. 2020 Jan 9;17(2):449. doi: 10.3390/ijerph17020449 (PMC7013645; doi:10.3390/ijerph17020449)
Supplement: Supplementary file 1 [file ijerph-17-00449-s001.zip › ijerph-680901 - supplementary/Supplementary materials S1.docx]

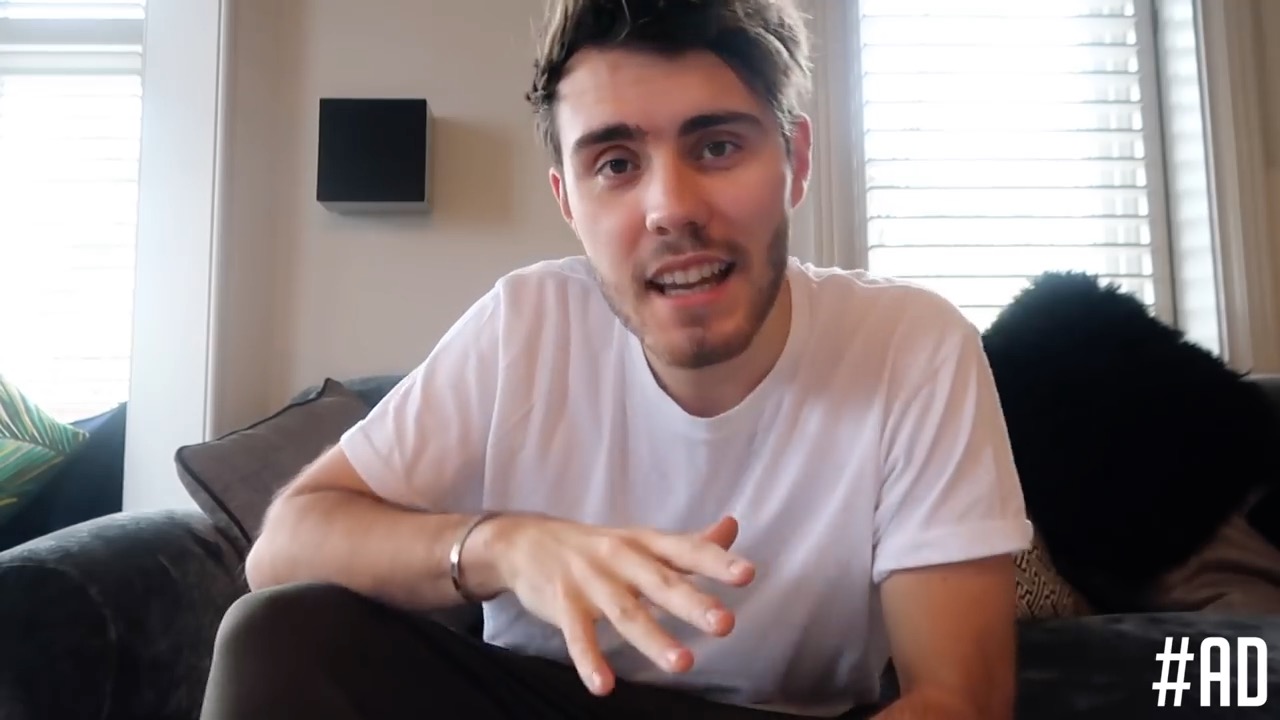


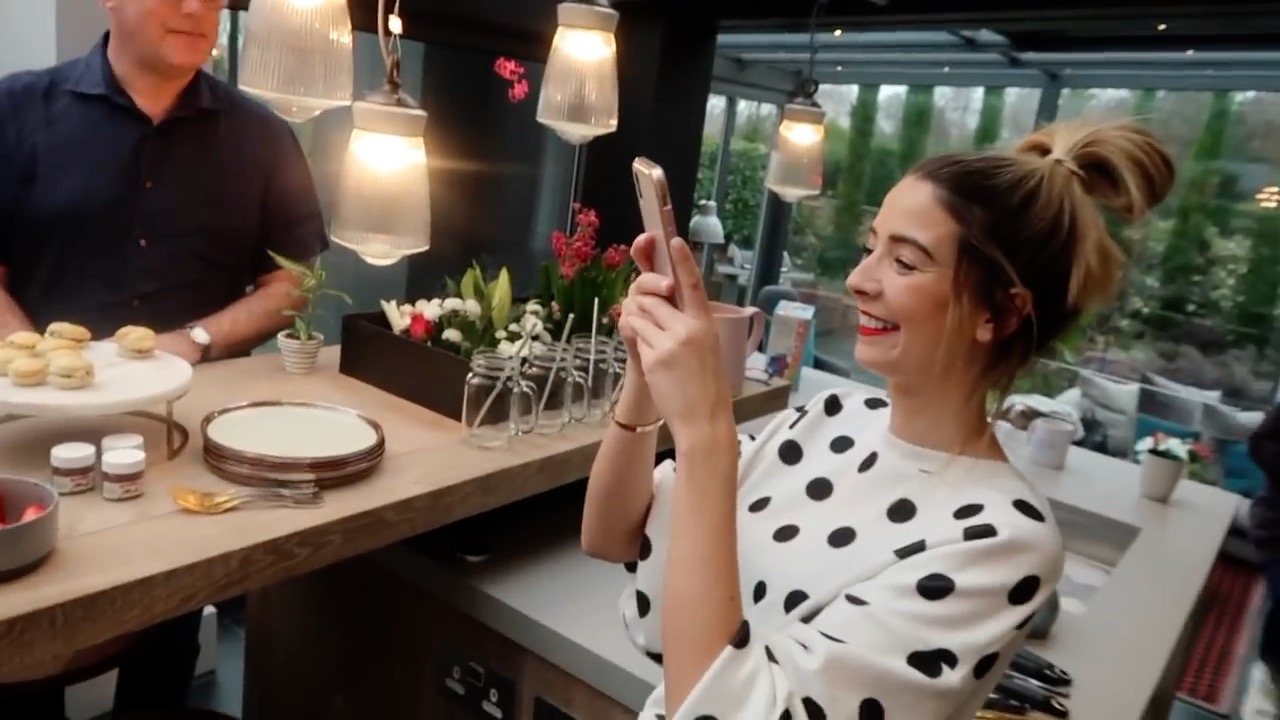


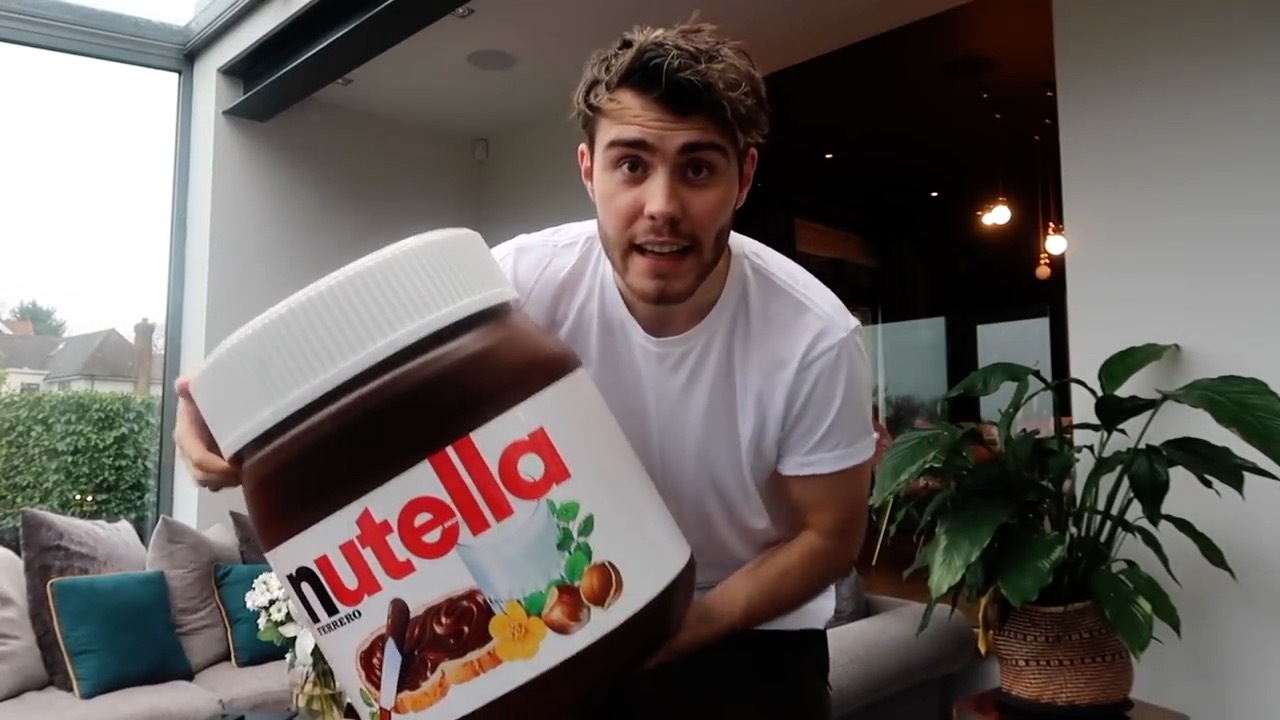


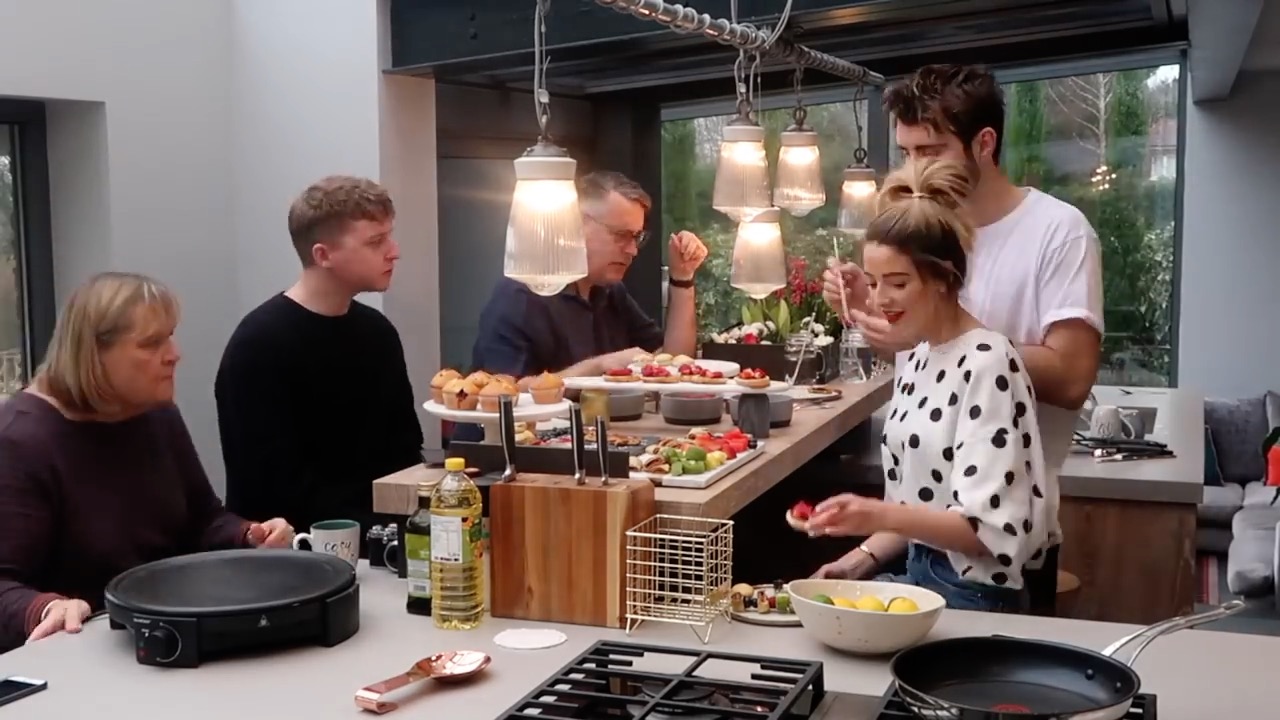


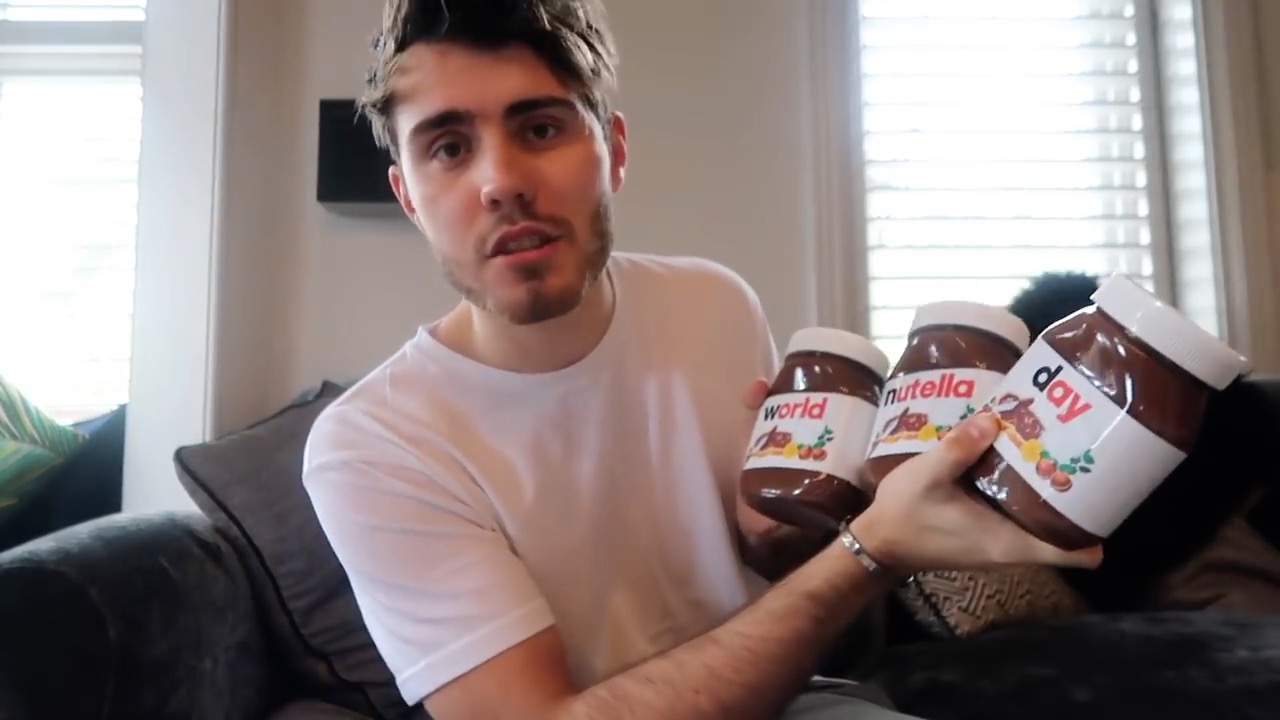


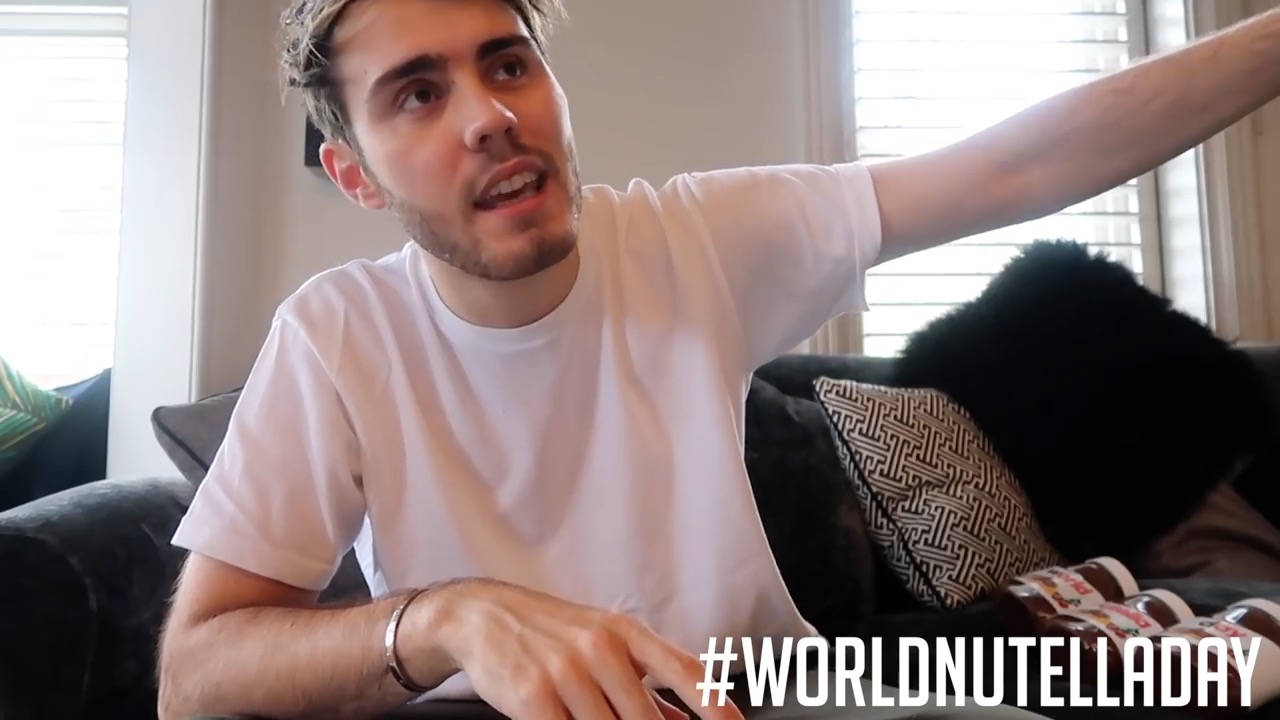


**Figure S1.** Photographic stills of influencer marketing techniques featured in the YouTuber’s video.
