# Supplementary material for: “It’s Just Addictive People That Make Addictive Videos”: Children’s Understanding of and Attitudes towards Influencer Marketing of Food and Beverages by YouTube Video Bloggers
Source: Int J Environ Res Public Health. 2020 Jan 9;17(2):449. doi: 10.3390/ijerph17020449 (PMC7013645; doi:10.3390/ijerph17020449)
Supplement: Supplementary file 1 [file ijerph-17-00449-s001.zip › ijerph-680901 - supplementary/Supplementary materials S2.docx]

Interview guide

Thank you for agreeing to help me today. My name is Anna and I am a researcher at the University of Liverpool who is interested in what you think about advertising on YouTube. In particular, I’m really interested to hear what you think about advertising by YouTube video bloggers, who you may know better as YouTubers.

YouTubers make videos that they share online with their viewers. Sometimes they show products (like foods or new phones) in their videos because they have been paid to do so, or because they have been sent the products by the company who makes them. This is a type of advertising. Today, we will watch a YouTube video that features advertising and afterwards I would like to ask what you thought about it.

I will be recording our conversation, so that I can remember what you said. I will take out your names so that when I write about your views no-one will know it was you that said them. Is that ok?

Can I ask everyone to not tell others about what is said in this group? This is so that everyone can say what they think without worrying about people knowing. Is that ok? There are no right or wrong answers today, I’m just interested in how *you* feel. Your opinions may not be the same as your friend, this is also fine. It would be nice for me to hear you discuss with each other why you have these thoughts.

Great, are we all ready to begin? Firstly, could I ask you to all write your name on the sticker provided. One at time I’d like you to say your names and ages please.

Next I would like to ask you some questions about YouTube and social media.

Section A—warm up

1. What is Instagram/Twitter/YouTube?
2. How often do you use YouTube?
3. Do you watch videos of YouTubers?
   1. If you had a friend who didn’t know what a YouTuber was, how would describe what they do?
4. Why do you watch/not watch YouTubers?
5. How does your favorite YouTuber compare to you?

*Check that all children have answered and prompt any if needed. Also be sure to know if anyone doesn’t use YouTube/watch YouTubers.*

Section B—video and laminates of marketing techniques

Great, it seems like you all know a bit about YouTubers. What we will do now is watch a video of a popular YouTuber. If you don’t recognise him, don’t worry. All you need to know is that he is called Alfie and he has a lot of subscribers. In the video there is an advert for Nutella chocolate spread. Alfie also talks about a day of the year that celebrates Nutella which is called World Nutella Day.

1. Who has heard of Nutella chocolate spread?
2. What do you think of Nutella?
3. Who has heard of World Nutella Day?
   1. Do any of you celebrate this day with family or friends?
   2. Why/How do you celebrate it?

I’m going to play the video now, is everyone ready? Please pay attention to it and don’t talk while it’s on because we will be talking about it afterwards.

PLAY VIDEO

1. Does anybody follow Alfie on social media?
2. What did you think about the video?
3. What sort of person do you think would enjoy watching this video?

To help you remember particular parts of the video, I am going to show you some photos. I’d like us to have a chat about these parts of the video. Remember there are no right and wrong answers, I just want to know what you think. Feel free to respond to what one another says.

At the start of the video we saw the YouTuber sitting down and talking to his viewers.

Show picture 1

1. What does the message in the bottom right hand side of the screen mean? (If no child responds with ‘advertising/advert’, then inform the group in simple terms)
   1. How does knowing the video was an advert make you feel?
   2. Have you seen YouTubers who advertise before?

A bit later in the video the YouTuber talks about a competition. To win the competition, viewers need to Tweet or Instagram the YouTuber using the hashtag ‘World Nutella Day’

Show picture 2

1. Why do you think the YouTuber’s video includes a competition?
2. What happens if the YouTuber’s viewers share the hashtag ‘World Nutella Day’ on social media?

When the YouTuber plays the piano in the video he shows his viewers the seat that Nutella gave him as a present.

Show picture 3

1. Why do you think the YouTuber was given this present from Nutella?

In the video, when all the food is ready, the YouTuber and his family take photos of it before they start eating.

Show picture 4

Why do you think that the YouTuber’s girlfriend Zoe (who is also a YouTuber), wants to share her photos of the food on Instagram?

Next in the video, the YouTuber and his family all sit down together eat the foods.

Show picture 5

How did you feel when all the family got together to eat the foods?

- 1. What foods do you remember seeing?
  2. Would your family eat Nutella like this at home?

1. Do you think the YouTuber and his family like to eat Nutella?
2. The brand name ‘Nutella’ is said a lot in the video. Why do you think this is?

At the end of the video, the YouTuber asked his viewers to share their Nutella recipes, pictures and videos with him on Twitter and Instagram using the hashtag ‘World Nutella Day’.

Show picture 6

Why does the YouTuber want his viewers to do this?

1. The YouTuber said he would reply to viewers who shared their pictures and videos with him on social media.
   1. How would it make you feel if your favorite YouTuber replied to you because of something you shared?
   2. Is a YouTuber a celebrity?

Section C—children’s overall impressions

Thank you for your help so far. I would like to ask you a few more questions now about how you feel about advertising.

1. How do you feel about Nutella after watching this advert?
2. Would the YouTuber have made the advert for Nutella if he didn’t like Nutella?
3. Is it important to you that the YouTuber really does like Nutella—why?
4. What do you think about this sort of advertising?
5. Can you tell me about another time you have seen a YouTuber advertise something?
6. How do you feel about this type of advertising compared to other types of advertising?
7. Can we trust YouTubers to give us correct information about products?

We have come to the end of all the questions. Thank you very much for sharing your thoughts and feelings about the video we watched today. Does anybody have anything else they would like to say?

DEBRIEF
